# Stochastic excitation for high-resolution atomic force acoustic microscopy imaging: a system theory approach

**DOI:** 10.3762/bjnano.11.58

**Published:** 2020-05-04

**Authors:** Edgar Cruz Valeriano, José Juan Gervacio Arciniega, Christian Iván Enriquez Flores, Susana Meraz Dávila, Joel Moreno Palmerin, Martín Adelaido Hernández Landaverde, Yuri Lizbeth Chipatecua Godoy, Aime Margarita Gutiérrez Peralta, Rafael Ramírez Bon, José Martín Yañez Limón

**Affiliations:** 1 Universidad Cuauhtémoc. Blvd. Bernardo Quintana A. #229. Fracc. Los Arcos C.P. 76060, Querétaro, Querétaro, México; 2 CINVESTAV. Libramiento Norponiente #2000 C.P. 76230, Fracc. Real de Juriquilla, Querétaro, Querétaro, México; 3 CONACYT-Facultad de Ciencias Físico-Matemáticas, Benemérita Universidad Autónoma de Puebla, Av. San Claudio y Av. 18 sur, Col. San Manuel Ciudad Universitaria, C.P. 72570, Puebla, Puebla, México; 4 Facultad de Ciencias Físico Matemático, Universidad Autónoma del Estado Chiapas, Carr. Emiliano Zapata Km 8 Tuxtla Gutiérrez, Chiapas C.P. 29050, México; 5 Centro de Investigación Científica y de Educación Superior de Ensenada, Baja California. Carretera Ensenada - Tijuana No. 3918, Zona Playitas, CP. 22860, Ensenada, B.C. México; 6 Centro de Nanociencias y Nanotecnología, UNAM, Km 107 Carretera Tijuana-Ensenada C.P. 22800. Ensenada, Baja California, México; 7 Departamento de Minas, Metalurgía y Geología, Universidad de Guanajuato. Ex Hacienda San Matías s/n C.P. 36020. Guanajuato, Guanajuato, México

**Keywords:** atomic force microscopy, fast Fourier transform, mechanical properties, system theory, white noise

## Abstract

In this work, a high-resolution atomic force acoustic microscopy imaging technique is developed in order to obtain the local indentation modulus at the nanoscale level. The technique uses a model that gives a qualitative relationship between a set of contact resonance frequencies and the indentation modulus. It is based on white-noise excitation of the tip–sample interaction and uses system theory for the extraction of the resonance modes. During conventional scanning, for each pixel, the tip–sample interaction is excited with a white-noise signal. Then, a fast Fourier transform is applied to the deflection signal that comes from the photodiodes of the atomic force microscopy (AFM) equipment. This approach allows for the measurement of several vibrational modes in a single step with high frequency resolution, with less computational cost and at a faster speed than other similar techniques. This technique is referred to as stochastic atomic force acoustic microscopy (S-AFAM), and the frequency shifts of the free resonance frequencies of an AFM cantilever are used to determine the mechanical properties of a material. S-AFAM is implemented and compared with a conventional technique (resonance tracking-atomic force acoustic microscopy, RT-AFAM). A sample of a graphite film on a glass substrate is analyzed. S-AFAM can be implemented in any AFM system due to its reduced instrumentation requirements compared to conventional techniques.

## Introduction

There are several methods to measure mechanical properties at the nanoscale level, based on, e.g., nanoindentation or on other physical phenomena [1–2]. However, each method has its limitations due to instrumentation capabilities and the geometry of the contact. Also, some of these methods can be destructive or provide only poor resolution because of the nanometric dimensions [1]. Atomic force microscopy (AFM) is a fundamental tool in nanotechnology [3] because it offers a non-destructive alternative for measuring mechanical properties at the nanoscale using the small size of the cantilever tip with a radius of 5–50 nm.

There are two kinds of conventional AFM methods for the measurement of mechanical properties [4–5], i.e., the measurement of force–displacement curves or of contact resonance frequencies. The techniques based on force–displacement curves are ideal when the stiffness of the cantilever and the sample are similar. The techniques based on contact resonance frequencies are appropriate when the stiffness of the sample material is larger than the cantilever stiffness. When the tip is out of contact, the resonance modes occur at specific frequencies, which depend on the geometrical and material properties of the cantilever. And when the tip touches the sample material, the frequencies of the resonance modes increase due to tip–sample interaction. The frequency shifts can be used with a suitable model to calculate the mechanical properties of the sample material. This can be achieved by an external actuator or by an actuator attached to the cantilever holder chip [1,6–11].

The methods that use the resonance frequencies are often labeled as acoustic or ultrasonic methods due to the frequency range of the vibrations involved (from 100 kHz to 3 MHz) [1,9,12]. Among them are ultrasonic force microscopy (UFM) [13], heterodyne force microscopy [14], ultrasonic atomic force microscopy (UAFM), atomic force acoustic microscopy (AFAM) [1], bimodal AFM [15], resonance tracking-atomic force acoustic microscopy (RT-AFAM) [7], band excitation [10], dual-frequency resonance-tracking atomic force microscopy [16], nanomechanical spectroscopy [2], G-mode [17] and triple frequency atomic force microscopy [18]. Even though these methods offer reliable measurements, they can only measure one or three resonant vibrational modes with a relative frequency resolution, and in some cases, the involved instrumentation can be very complex [2,10,16–17,19]. This makes the system excitation restricted to purely sinusoidal signals for a measurement based on a lock-in amplifier. When a lock-in amplifier is used, it reduces the time response of the measurement process [10,17,20].

In this work, an AFM technique is presented that is based on resonance frequency shifts. The main advantages of this technique are reduced instrumentation requirements and higher frequency resolution at different resonant modes. Also, more than one vibrational mode in each measurement step as well as indentation modulus mappings are obtained. This is possible when system theory [21] is taken into account, i.e., the system identification problem [22]. Here, a mathematical model that describes the resonance frequencies for a free cantilever and a cantilever at contact with the sample is calculated for stochastic perturbations of the tip–sample interaction. For this reason, the technique is referred to as stochastic atomic force acoustic microscopy (S-AFAM).

S-AFAM works as follows: While a conventional AFM is scanning in contact mode, the tip–sample interaction is excited by a white-noise signal generated by a function waveform generator through a piezoelectrical actuator. At the same time, a fast Fourier transform (FFT) is computed by data acquisition equipment using the deflection signal. Each FFT spectrum corresponds to one pixel of the sample and is stored on a hard disk drive. This way of measurement enhances the frequency window for analysis and does not require a lock-in amplifier, which reduces the time response of the overall measurement. At the end of the scanning, all the acquired FFT spectra yield a 128 × 128 pixels mapping in which the resolution for frequency shifts is about 153.8 Hz. Then, an offline process is carried out using a software routine in Matlab, which is based on a mathematical model that relates the contact resonance frequencies with an indentation modulus value. This model is based on the power spectral density (PSD) and the harmonic oscillator model. At the end of the process, an indentation modulus mapping is obtained.

This paper is organized as follows: in the section “Instrumentation Setup”, the prototype is described with further details, including the acquisition data process and the AFM equipment. Then, in the section “Mathematical Model”, the mathematical background for the power spectral density is described for a free cantilever and a cantilever in contact with the sample when white-noise excitation is taken into account. After that, in the “Results and Discussion” section, the obtained images using S-AFAM are explained with further details and compared to RT-AFAM results. Finally, we end with conclusions about the capabilities of the technique.

## Instrumentation Setup

**Figure 1:**
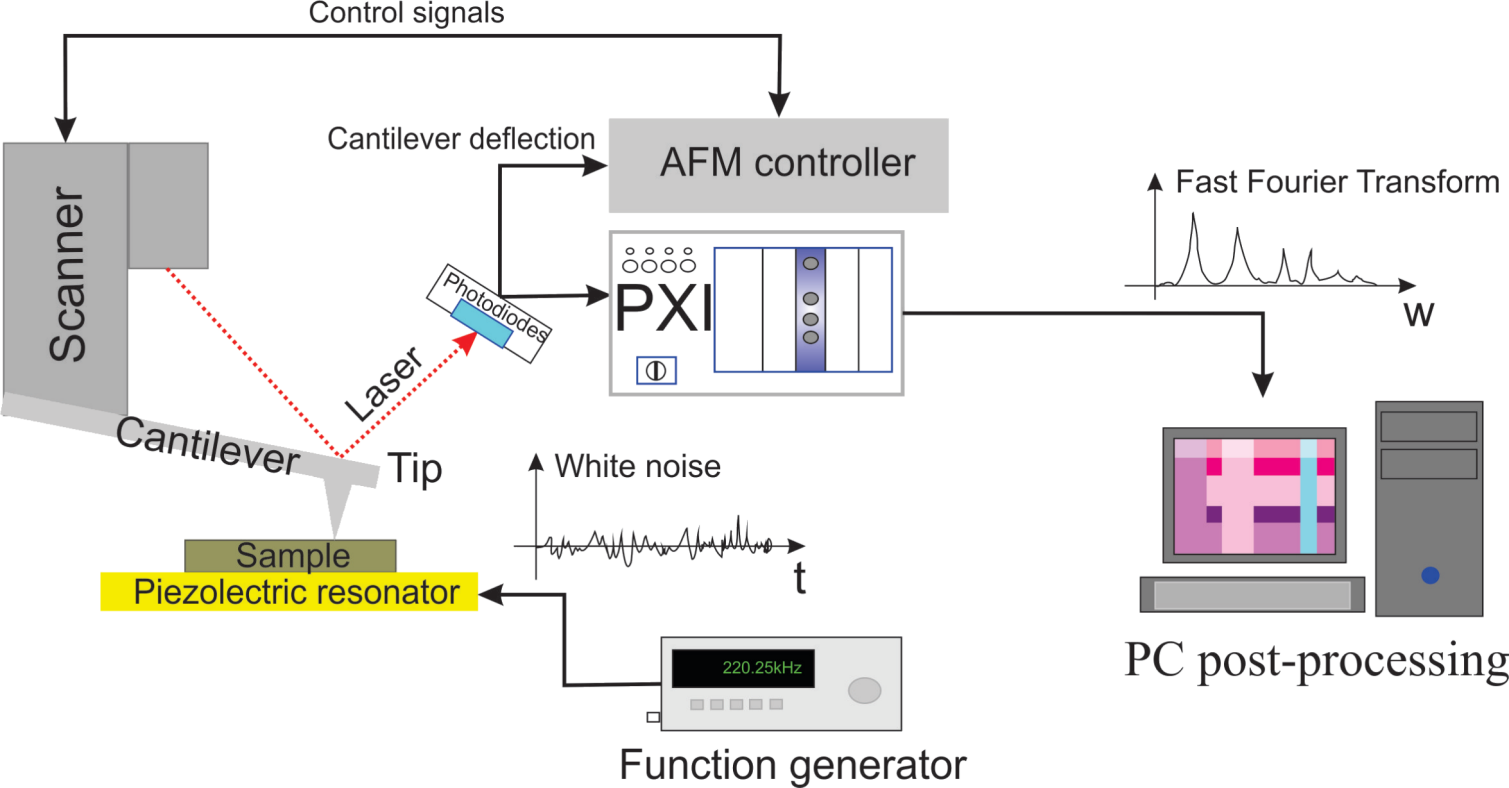
Experimental setup for S-AFAM, using a NI PXIe-1073 device and a function waveform generator HP/Agilent 33120A.

The S-AFAM instrumentation setup is connected to commercial AFM equipment (Figure 1). The following list gives a detailed description of the instrumentation:

- A SPM, Bruker / Veeco / Digital Instruments Nanoscope IV Dimension 3100 device was used, which was upgraded with a closed-loop *x*–*y* nanopositioning stage (nPoint, Inc. NPXY100) and a signal access module (SAM) used for signal input/output to the AFM. The apparatus was supported on a floating air table and equipped with an acoustical isolation chamber, which minimize the external thermal and vibrational disturbances, respectively.
- Data acquisition and FFT processing were carried out using a NI PXIe-1073 device, which includes a NI 7961R FPGA, a NI 5762 digitizer at 200MS/s/ch and a PXI 6363DAQ from National Instruments.
- The white-noise signal is generated by a function waveform generator HP/Agilent 33120A.
- BudgetSensors diamond-coated silicon probes, 450 µm long with a spring constant of 0.2 N/m were used.
- All experiments were carried out in dry air at a temperature of 21.0 ± 0.1 °C and a relative humidity of (2 ± 1)%.

It is very important to define the appropriate signal to perturb the system. This allows for gathering sufficient information about the system dynamics. For this work, a stochastic signal is used for the tip–sample excitation because it can extract all the system dynamics, i.e., persistent excitation in the system theory field [23–24].

A FFT of the deflection signal from the photodiodes is computed by the NI PXIe-1073 device. One FFT is carried out for each specific point of the sample during a conventional AFM scanning of 128 × 128 pixels. While the system is executing this task, the HP/Agilent 33120A is exciting the tip–sample system through an external piezoelectric actuator below the sample using a white-noise signal. The white-noise approximation is used for this purpose because it can excite the tip–sample system using a 10 MHz flat-bandwidth signal [21–25].

**Figure 2:**
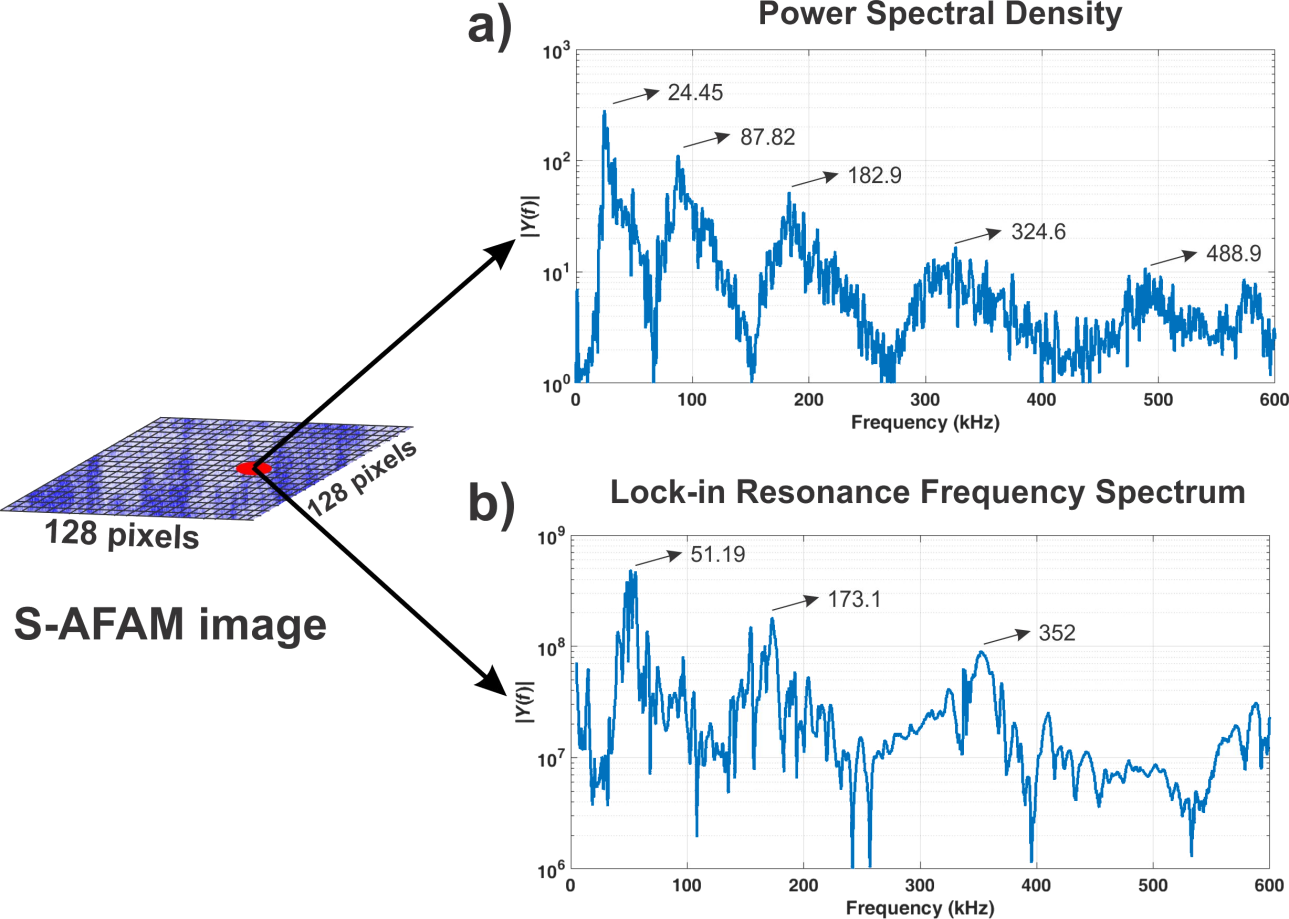
Contact resonance frequencies for a graphite film on a glass substrate. a) Resonance flexural modes acquired using S-AFAM, b) resonance flexural modes acquired using a lock-in amplifier.

The FFT spectra obtained from the 128 × 128 pixels mapping are stored on a hard disk drive. Subsequently, offline processing is carried out for each FFT spectrum using a harmonic oscillator model fit in which each pixel has an FFT spectrum with at least four resonance frequencies (Figure 2). In this manner, the FFT spectra are transformed into an indentation modulus mapping using a mathematical model based on the reduced elasticity modulus and the PSD model for a free cantilever and a cantilever in contact with the sample. The PSD is used because it is the ideal tool for treating the stochastic process in the frequency domain [26].

This way of signal enhancement allows for the measurement of several resonance frequencies in one single step and without a lock-in amplifier (Figure 2). To show the capabilities of this technique, a graphite film was sputtered on a glass substrate and characterized by the proposed S-AFAM technique and by conventional RT-AFAM [7].

## Mathematical Model

### Dynamic model

In order to extract the resonance frequencies of a free cantilever and a cantilever in contact with the sample, the model by Vázquez et al. [27–29] was used and then applied to a PSD model to transform the resonance frequencies to indentation modulus values. This model is necessary because the white-noise signal belongs to the power signals set. That is, these signals offer infinite energy [25–26].

**Figure 3:**
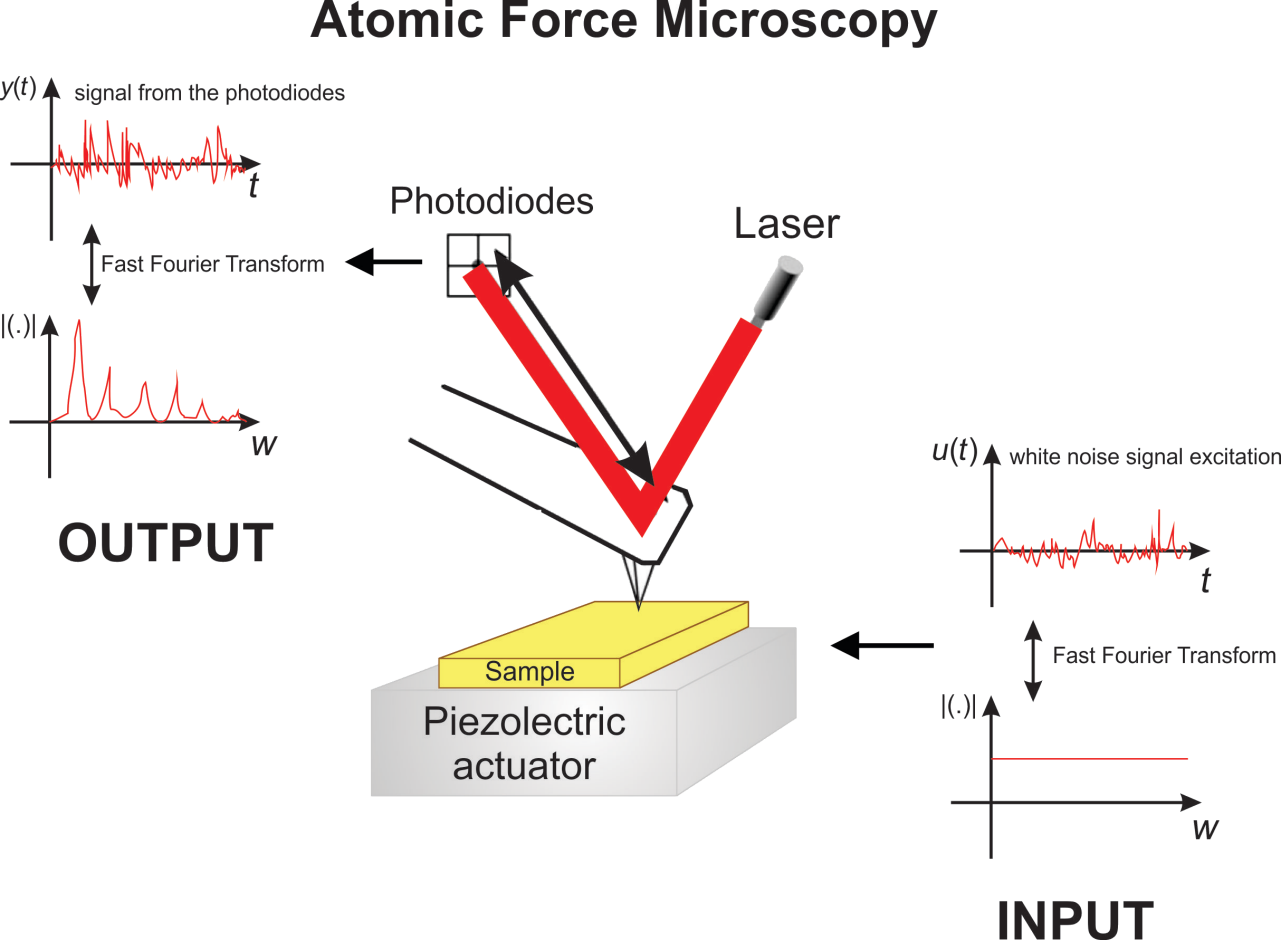
AFM system, the piezoelectrical signal excitation is considered to be the input, while the deflection signal from the photodiodes is considered to be the output.

In this work, the tip–sample interaction must be studied from the point of view of system theory [21,23] (Figure 3). The input system is the excitation signal through the piezoelectrical actuator, which can be controlled in amplitude and frequency. And the output system is the deflection signal from the photodiodes of the AFM equipment.

The classical Euler–Bernoulli beam equation is used, which is expressed by Vázquez et al. as [27–29]:

[1] $$EI\frac{\partial^{4}z\left(x,t\right)}{\partial x^{4}}+c\frac{\partial z\left(x,t\right)}{\partial t}+m\frac{\partial^{2}z\left(x,t\right)}{\partial t^{2}}=-u\left(t\right),$$

where *EI* is the flexural stiffness, *c* is the damping due to viscous friction, *m* is the mass per unit length and *z*(*x*,*t*) is the deflection of the cantilever defined for a displacement toward the sample, *t* is the time, *x* ∈ [0,*L*], *u*(*t*) is a force per unit length acting along the cantilever and *L* is the length of the cantilever. The boundary conditions at the fixed end are

[2] $$\begin{array}{c} {z\left(x,t\right)|}_{x=0}=0,\\ {\frac{\partial z\left(x,t\right)}{\partial x}|}_{x=0}=0, \end{array}$$

and at the tip end they are

[3] $$\begin{array}{c} {\frac{\partial^{2}z\left(x,t\right)}{\partial x^{2}}|}_{x=L}=0,\\ {EI\frac{\partial^{3}z\left(x,t\right)}{\partial x^{3}}|}_{x=L}+f\left(t\right)=-q\left(t\right), \end{array}$$

where *q*(*t*) is the input force acting perpendicular to the cantilever and *f*(*t*) is the interaction force between the cantilever and the surface expressed by the Derjaguin–Muller–Toporov (DMT) model [1] as

[4] $$f\left(t\right)=-\frac{HR}{6a_{0}^{2}}+\frac{4}{3}E^{*}\sqrt{R}{\left(z_{s}-z\left(x,t\right)+a_{0}\right)}^{3/2}.$$

Here, *H* is the Hamaker constant, *R* is the tip radius, E* is the reduced elastic modulus between the tip and the sample, *a*_0_ is the interatomic distance and *z*_s_ is the distance from the sample to the tip of the undeflected cantilever, which is described by the force *f*(*t*) linearized around a point *z*_0_ as

[5] $$\begin{array}{c} f\left(t\right)=-{\frac{\partial f\left(t\right)}{\partial z\left(L,t\right)}|}_{z_{0}}z\left(x,t\right)\\ =-2E^{*}\sqrt{R\left(z_{s}-z_{0}+a_{0}\right)}\left(z\left(x,t\right)\right). \end{array}$$

In this equation 
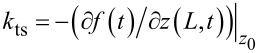
 represents the contact stiffness. Then, the linearized model around *z*_0_ according to Equation 3 is

[6] $$EI\left({\frac{\partial^{3}z\left(x,t\right)}{\partial x^{3}}|}_{x=L}-\frac{{\hat{k}}_{\text{ts}}}{3L^{3}}z\left(x,t\right)\right)=-q\left(t\right),$$

where

[7] $$k_{\text{ts}}=\frac{3EI}{L^{3}}{\hat{k}}_{\text{ts}}.$$

Using the boundary conditions, the Laplace transform is applied to Equation 1. In this way, two transfer functions can be obtained as follows [27–29]:

1. The transfer function for the free cantilever is

[8] $$\begin{array}{c} G_{\text{free}}\left(x,s\right)={\frac{Z\left(x,s\right)}{U\left(s\right)}|}_{q=0}\\ =-2\frac{\cosh\left(\lambda x\right)\sin\left(\lambda x\right)-\cos\left(\lambda x\right)\sinh\left(\lambda x\right)}{EI\lambda^{3}\left(4+2\cos\left(2\lambda x\right)+2\cosh\left(2\lambda x\right)\right)}. \end{array}$$

This expression takes into account a distributed uniform force acting along the cantilever. This force is caused by the piezoelectric actuator below the cantilever chip.

2. The transfer function for cantilever in contact with the sample is

[9] $$\begin{array}{c} G_{\text{cont}}\left(x,s\right)={\frac{Z\left(x,s\right)}{Q\left(s\right)}|}_{u=0}\\ =\frac{\sinh\left(2\lambda x\right)-\sin\left(2\lambda x\right)}{EI\lambda^{3}\left(4+2\cos\left(2\lambda x\right)+2\cosh\left(2\lambda x\right)\right)}. \end{array}$$

This expression takes into account the force acting at the tip end of the cantilever. This makes Equation 9 suitable when the tip is in contact with the sample material.

Also,

[10] $$\lambda \left(s\right)=\sqrt[{4}]{\frac{cs+ms^{2}}{4EI}}.$$

Now, Equation 8 and Equation 9 have to be considered in a mathematical process as follows: (1) Each numerator and each denominator, either of Equation 8 or of Equation 9, have to be changed to a matrix form. (2) Once each numerator is in a matrix form, the PSD formula is applied to this matrix form. (3) Once each denominator is in a matrix form, the PSD formula is applied to this matrix form. (4) The PSD for each numerator and denominator are put together in one equation, which is the same transfer function described in either Equation 8 or Equation 9, but in the frequency domain as a result of the stochastic excitation and the linearization process.

### Free cantilever transfer function

For the free cantilever, when it is excited by white-noise, Equation 8 has to be considered as a transfer function using a PSD treatment. This transfer function describes the relationship between the excitation with the piezoelectric actuator, which is below the cantilever chip, and the cantilever deflection according to the system described in Figure 3. This is expressed using some equalities [30] as follows:

[11] $$G_{\text{free}}\left(x,s\right)=\frac{Z\left(x,s\right)}{U\left(s\right)}=\frac{-4L^{3}\prod_{n=1}^{\infty }\left[1-\frac{\lambda^{4}L^{4}}{n_{n}^{4}}\right]}{24EI\prod_{n=1}^{\infty }\left[1+\frac{\lambda^{4}L^{4}}{d_{n}^{4}}\right]},$$

where *n_n_* and *d_n_* are the *n*-th roots of


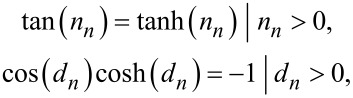


respectively.

Thus, Equation 11 is expanded using Equation 10 as

[12] $$\begin{array}{c} G_{\text{free}}\left(x,s\right)=\frac{Z\left(x,s\right)}{U\left(s\right)}\\ =\left(\frac{-4L^{3}}{3EI}\right)\left[\frac{\prod_{n=1}^{\infty }\left(\frac{-mL^{4}}{4n_{n}^{4}}s^{2}-\frac{c}{4n_{n}^{4}}s+\frac{EI}{L^{4}}\right)}{\prod_{n=1}^{\infty }\left(\frac{m}{d_{n}^{4}}s^{2}+\frac{c}{d_{n}^{4}}s+\frac{EI}{L^{4}}\right)}\right]. \end{array}$$

Now, the PSD has to be computed from Equation 12 since the system is excited by a stochastic signal [25–26]. For this work, a white-noise signal is considered, because it features an infinitely flat bandwidth. White noise is defined as a scalar second-order discrete-time stochastic process for a voltage generated by the function waveform generator, *V_(−∞,∞)_*, and its properties are


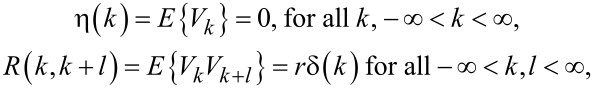


where *r* ≥ 0, the mean *E*{*V_k_*} is the expected value of the random variable *V*(*k*), the autocorrelation *E*{*V_k_V_k+l_*} is the expected value of the product *V_k_V_k+l_*, and δ(*k*) is the Dirac delta function [25–26,31].

Then, a PSD must be calculated for each numerator and denominator. The PSD for a denominator term is calculated taking into account the *n*-th denominator from Equation 12 as


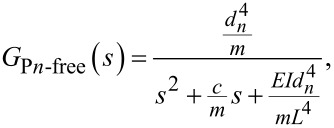


which can be transformed into the matrix form [21,29] as

[13]
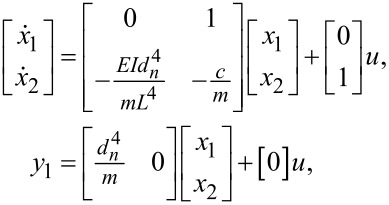


where *x*_1_ is the deflection of the cantilever, 
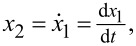
 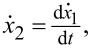 *y*_1_ is the deflection of the cantilever, and *u* is the force described in Equation 1.

The PSD model [25] for a second-order system is

[14] $$G_{yy}\left(\omega \right)=C{\left(-j\omega I-A\right)}^{-1}BG_{\omega \omega }\left(\omega \right)B^{T}{\left(j\omega I-A\right)}^{-T}C^{T},$$

where ω is the frequency,

[15] $$A=\left[\begin{array}{cc} 0 & 1\\ -\frac{EId_{n}^{4}}{mL^{4}} & -\frac{c}{m} \end{array}\right],\;B=\left[\begin{array}{c} 0\\ 1 \end{array}\right],\;C=\left[\begin{array}{cc} \frac{d_{n}^{4}}{m} & 0 \end{array}\right],$$

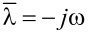
 as the complex conjugate of λ, the white-noise power is

[16] $$E\left[\omega \left(t\right)\omega \left(\tau \right)\right]=V\delta \left(t-\tau \right),\;BG_{\omega \omega }\left(\omega \right)B^{T}=\left[\begin{array}{cc} 0 & 1\\ 0 & V \end{array}\right],$$

and *V* is the voltage amplitude for the white-noise signal. Thus, Equation 14 for the *n*-th denominator becomes

[17] $$G_{\text{P}yy\text{-free}}\left(\omega \right)=\frac{\frac{Qd_{n}^{8}}{m^{2}}}{{\left(\omega^{2}-\frac{EId_{n}^{4}}{mL^{4}}\right)}^{2}+\frac{c^{2}}{m^{2}}\omega^{2}}.$$

Now, the PSD for the *n*-th numerator is calculated taking into account Equation 12 as:


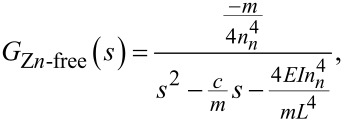


which can be transformed into the matrix as

[18]
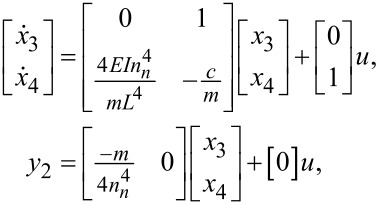


where *x*_3_ is the deflection of the cantilever, 
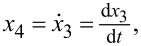
 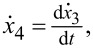 *y*_2_ is the deflection of the cantilever, and *u* is the force described in Equation 1.

Using the same formula described in Equation 14 for the PSD and the equalities

[19] $$A^{\prime }=\left[\begin{array}{cc} 0 & 1\\ \frac{4EIn_{n}^{4}}{mL^{4}} & -\frac{c}{m} \end{array}\right],\;B^{\prime }=\left[\begin{array}{c} 0\\ 1 \end{array}\right],\;C^{\prime }=\left[\begin{array}{cc} \frac{-m}{4n_{n}^{4}} & 0 \end{array}\right],$$

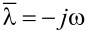
 is the complex conjugate of λ, and the white-noise power is described in Equation 16. The PSD for the *n*-th numerator becomes

[20] $$G_{\text{Z}yy\text{-free}}\left(\omega \right)=\frac{\frac{Qm^{2}}{16n_{n}^{8}}}{{\left(\omega^{2}+\frac{4EIn_{n}^{4}}{mL^{4}}\right)}^{2}+\frac{c^{2}}{m^{2}}\omega^{2}}.$$

Finally, taking into account Equation 17 and Equation 20, the PSD for Equation 11 during excitation with white noise is

[21] $$G_{\text{free}}\left(\omega \right)=\frac{-4L^{3}}{3EI}\frac{\prod_{n=1}^{\infty }\left[\frac{{\left(\omega^{2}+\frac{4EIn_{n}^{4}}{mL^{4}}\right)}^{2}+\frac{c^{2}}{m^{2}}\omega^{2}}{\frac{Qm^{2}}{16n_{n}^{8}}}\right]}{\prod_{n=1}^{\infty }\left[\frac{\frac{Qd_{n}^{8}}{m^{2}}}{{\left(\omega^{2}-\frac{EId_{n}^{4}}{mL^{4}}\right)}^{2}+\frac{c^{2}}{m^{2}}\omega^{2}}\right]}.$$

This equation could be obtained once the model was linearized. The PSD can be calculated for each denominator and numerator in the frequency domain independently [21,26,32]. And, using this PSD, Equation 21 is obtained, which describes a transfer function for the free cantilever in the frequency domain when it is excited by a stochastic signal.

### Transfer function for a cantilever in contact

The transfer function is defined from Equation 9 as

[22] $$G_{\text{cont}}\left(x,s\right)=\frac{Z\left(x,s\right)}{U\left(s\right)}=\frac{\frac{8L^{3}}{3}\prod_{n=1}^{\infty }\left[1+\frac{4\lambda^{4}L^{4}}{n_{n}^{4}}\right]}{8EI\left(1+{\hat{k}}_{\text{ts}}\right)\prod_{n=1}^{\infty }\left[1+\frac{4\lambda^{4}L^{4}}{d_{n}^{4}}\right]},$$

where 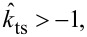 *n_n_* and *d_n_* are the *n*-th roots of


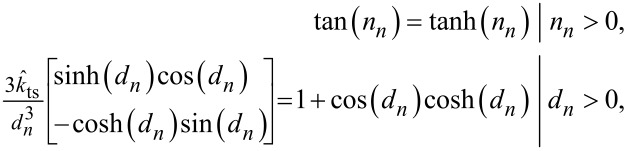


respectively.

Now, using the same methodology as in the last section to obtain the transfer function for a free cantilever excited by white-noise [30], the PSD for a cantilever in contact with the sample is obtained. From Equation 22 the *n*-th denominator is described by

[23] $$G_{\text{P}n\text{-cont}}\left(s\right)=8EI\left(1+{\hat{k}}_{\text{ts}}\right)\left[1+\frac{4\lambda^{4}L^{4}}{d_{n}^{4}}\right].$$

Using Equation 14 and Equation 23, the PSD for the *n*-th denominator is calculated as

[24] $$G_{\text{P}yy\text{-cont}}\left(\omega \right)=\frac{\frac{Qd_{n}^{8}}{64{\left(1+{\hat{k}}_{\text{ts}}\right)}^{2}L^{8}m^{2}}}{{\left(\omega^{2}-\frac{EId_{n}^{4}}{mL^{4}}\right)}^{2}+\frac{c^{2}}{m^{2}}\omega^{2}}.$$

Also from Equation 22, the *n*-th numerator is described by

[25] $$G_{\text{Z}n\text{-cont}}=\frac{8L^{3}}{3}\left[1+\frac{4\lambda^{4}L^{4}}{n_{n}^{4}}\right].$$

Using Equation 14 and Equation 25, the PSD for the *n*-th numerator is calculated as

[26] $$G_{\text{Z}yy\text{-cont}}\left(\omega \right)=\frac{{\left(\omega^{2}-\frac{EIn_{n}^{4}}{mL^{4}}\right)}^{2}+\frac{c^{2}}{m^{2}}\omega^{2}}{\frac{9QE^{2}I^{2}n_{n}^{8}}{m^{2}L^{8}}}.$$

Finally, using Equation 24 and Equation 26, the PSD for contact cantilever is calculated for Equation 22 as

[27] $$G_{\text{cont}}\left(\omega \right)=\frac{\prod_{n=1}^{\infty }\left[\frac{{\left(\omega^{2}-\frac{EIn_{n}^{4}}{mL^{4}}\right)}^{2}+\frac{c^{2}}{m^{2}}\omega^{2}}{\frac{9QE^{2}I^{2}n_{n}^{8}}{m^{2}L^{8}}}\right]}{\prod_{n=1}^{\infty }\left[\frac{\frac{Qd_{n}^{8}}{64{\left(1+{\hat{k}}_{\text{ts}}\right)}^{2}L^{8}m^{2}}}{{\left(\omega^{2}-\frac{EId_{n}^{4}}{mL^{4}}\right)}^{2}+\frac{c^{2}}{m^{2}}\omega^{2}}\right]}.$$

## Results and Discussion

In the literature, similar works give an indentation modulus for each resonance frequency, which leads to more than one value for the indentation modulus in the measurement. In this work, Equation 21 and Equation 27 are useful because these give a quantitative relationship between a set of resonance frequencies and an indentation modulus using white-noise excitation. The validation of this model requires simulations and measurements, which are presented and discussed.

**Figure 4:**
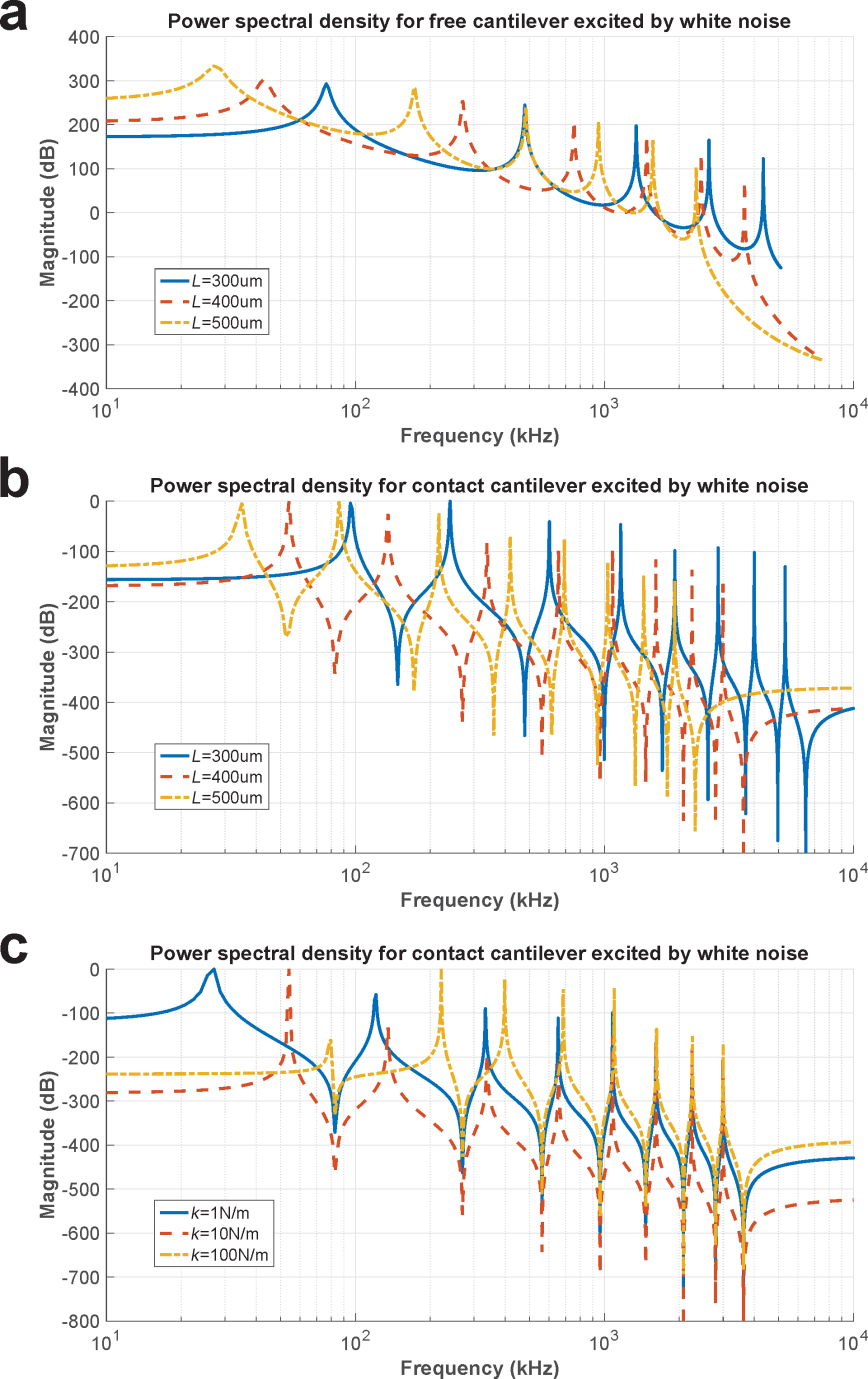
PSD simulation. a) Free cantilever: *L* = 300 μm(blue line), *L* = 400 μm (dashed red line) and *L* = 500 μm (dotted yellow line); b) cantilever in contact: *L* = 300 μm (blue line), *L* = 400 μm (dashed red line) and *L* = 500 μm (dotted yellow line); c) cantilever in contact for *L* = 400 μm: 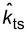 = 1 N/m (blue line), 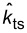 = 10 N/m (dashed red line), 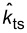 = 100 N/m (dotted yellow line).

Equation 21 and Equation 27 are simulated with the following numerical data obtained from [28]: *E* = 169.7 GPa, *I* = 3.64 × 10^−22^ m^4^, *c* = 1 × 10^−18^ kg/ms and *m* = 4.08 × 10^−7^ kg/m. For the free cantilever, the simulation is shown in Figure 4a. In this figure, a difference in resonance frequencies can be seen between a cantilever with *L* = 300 µm and another one with *L* = 500 µm. When the cantilever is shorter, the resonance frequencies increase. Using these computed free resonance frequencies, the geometry for a real cantilever can be known if a suitable routine is used to search for the cantilever that can be fitted best to these frequencies.

For a cantilever in contact with the sample, the simulation is shown for three cantilevers with a contact stiffness of 10 N/m and different lengths of *L* = 300, 400 and 500 µm (Figure 4b). The behavior of the resonance frequencies is similar to that of the free cantilever. When the cantilever is shorter, the resonance frequencies increase. This result indicates that the length of the cantilever must be taken into account for the sensitivity, because the range of frequencies depends on the local mechanical properties of the sample.

When a contact cantilever with *L* = 400 µm is used and its contact stiffness is changed, it can be seen how the resonance frequencies increase with increasing contact stiffness. Three simulations are shown for contact stiffness values of 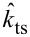 = 1, 10 and 100 N/m (Figure 4c).

The simulation results offer enough support for an experiment to show the capabilities of this technique. First, the geometrical parameters for the experimental cantilever must be known because the resonance frequency transformation into the indentation modulus requires these values. For this purpose, a resonance frequency spectrum was acquired for a free cantilever using white-noise as an excitation signal and the FFT algorithm. These resonance frequencies were fitted according to a database, which was computed using the free cantilever model described in Equation 21 and the particle swarm optimization algorithm [33–36], in order to obtain the geometrical parameters for the experimental cantilever. The database describes each cantilever according to length *L*, width *a*, thickness *b*, inertia moment *I* = *ab*^3^/12, linear mass *m* = ρ*A*, where *A* is the cross-sectional area of the cantilever and ρ = 2330 kg/m^3^ [37] is the density of the cantilever. The database has 10000 cantilevers where *L* ∈ [440,500] µm, *a* ∈ [40,50] µm, *b* ∈ [1,3] µm.

The optimization criteria are

[28] $$J=e_{\text{rms}}=\sqrt{\frac{1}{N}\sum_{n=1}^{N}{\left(f_{n}-{\hat{f}}_{n}\right)}^{2}},$$

where *e*_rms_ is the root mean square error, *f_n_* is the *n*-th measured free resonance frequency, and 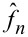 is the *n*-th theoretical free resonance frequency. For this work, using this optimization algorithm, the best results were obtained for a cantilever with the following dimensions: *L* = 460 µm, *a* = 58 µm, *b* = 1.8 µm.

In Table 1, the free resonance frequencies for the fitted experimental cantilever are compared to the experimental ones and to those obtained by using finite element analysis (FEA, [37]). There is an average error of 3.4% between the experimental frequencies and those obtained by using the proposed model, while there is a higher average error of approximately 5.6% between the experimental frequencies and those obtained by using FEA. Although there is an almost homogeneous error of 4% using the proposed model, it can provide a good approximation about the cantilever geometry using white-noise as an excitation signal. These results corroborate that S-AFAM is a suitable technique for the measurement of mechanical properties.

**Table 1:** Modeled and observed dynamic behavior for a free AFM cantilever.

Mode	Experiment (kHz)	FEA (kHz)	Error (%)	Model (kHz)	Error (%)

1	70.102	58.274	16.8	73.490	4.8
2	204.530	198.220	3.0	205.800	0.6
3	386.384	383.350	0.7	403.300	4.3
4	639.992	651.950	1.8	666.600	4.1

Also, the value of *k*_lever_ was calculated and compared with the fitted experimental cantilever used in this experiment. The comparison can be seen in Table 2. A commercial BudgetSensors diamond-coated silicon cantilever with 450 µm length and a spring constant of 0.2 N/m was considered. The first value was obtained from the manufacturer data, while the second value was obtained using the method by Sader [20], and the third value was obtained using *k*_lever_ = 3*EI*/*L*^3^, where the geometrical values were taken from the fit. It is important to notice that there is a good agreement between the Sader method and the proposed model, which makes S-AFAM a reliable method.

**Table 2:** Modeled values of *k*_lever_. A BudgetSensors diamond-coated silicon cantilever with 450 μm length and a spring constant of 0.2 N/m was used in this experiment.

Manufacturer *k*_lever_ (N/m)	Sader method *k*_lever_ (N/m)	Model *k*_lever_ (N/m)

0.2	0.179 ± 6.9%	0.1474 ± 3.4%

**Figure 5:**
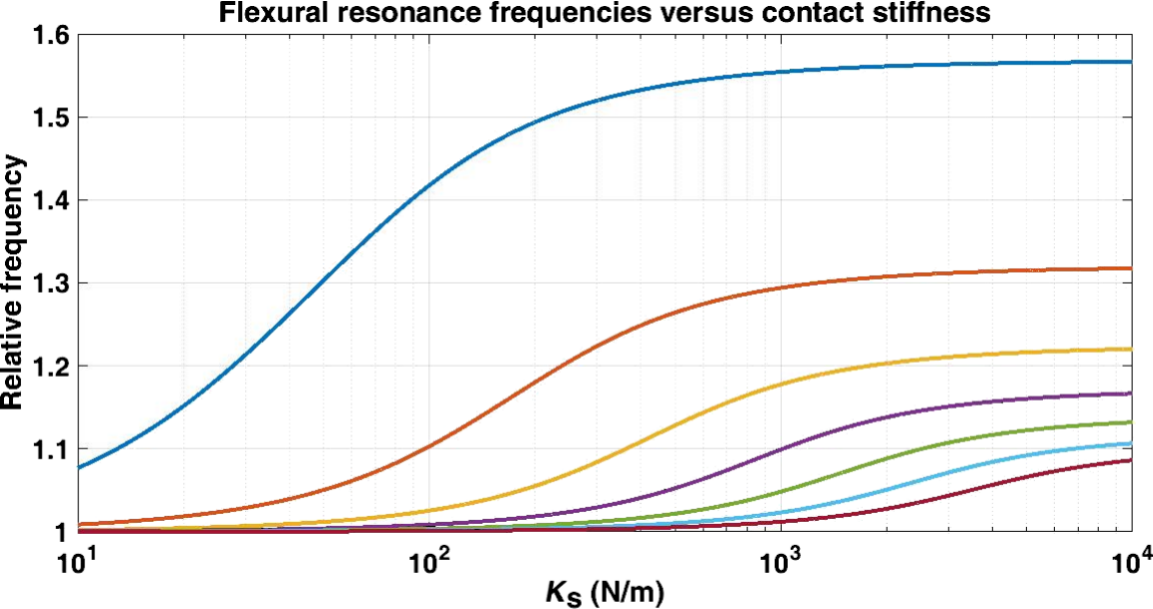
Flexural resonance frequencies as a function of the contact stiffness for a cantilever with the following geometrical parameters: *L* = 460 μm, *a* = 58 μm, *b* = 1.8 μm.

A conventional AFM mapping of a graphite film on a glass substrate was carried out using a white-noise signal as excitation of the tip–sample interaction. A FFT was computed for each pixel and stored on a hard disk drive. Figure 4c shows that the contact stiffness can be obtained from these contact resonance frequencies. It is important to notice that a set of resonance frequencies can provide a unique value for contact stiffness according to Equation 27 in a quantitative way. For this purpose, a mapping transformation from resonance frequencies to contact stiffness was obtained using a database for 8000 values for contact stiffness from 0.5 to 4000 N/m with a step of 0.5 N/m using the geometrical values for the cantilever obtained from the fit process (Figure 5).

**Figure 6:**
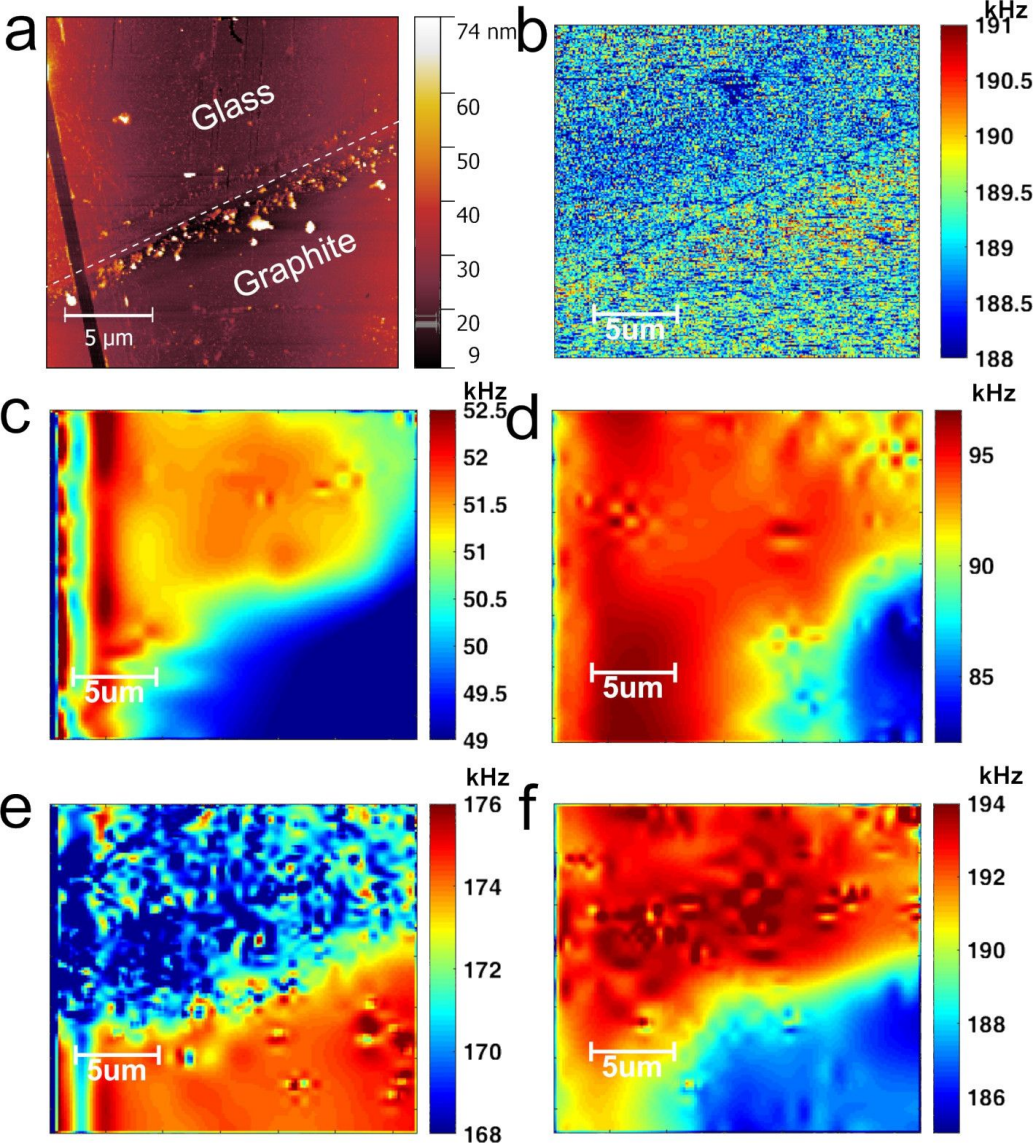
Results for a graphite film on a glass substrate. a) Conventional AFM topography; b) RT-AFAM for 188–191 kHz window; and S-AFAM frequency maps for c) 49–53 kHz window, d) 82–97 kHz window, e) 168–176 kHz window and f) 186–194 kHz window.

The measurement of a sputtered graphite film on a glass substrate shows the capabilities of S-AFAM. The thickness of the graphite film is 7 nm. A conventional AFM topography image is shown in Figure 6a. Film and substrate are labeled. Then, a comparison is shown between the results obtained by RT-AFAM and S-AFAM. Figure 6b shows an RT-AFAM image where the difference between materials is hardly noticeable.

S-AFAM was used to obtain images with higher frequency resolution, which makes the difference between graphite and glass easy to identify (Figure 6c–f). Also, when a window with higher frequencies is used, not only the difference between the two materials becomes clearer. Also, some details can be seen, which can be attributed to aggregates and imperfections of the deposited film. In Figure 6d, one of these details can be seen appreciated in the lower-left area where the resonance frequency contribution is higher for glass than for graphite. This result can be explained by imperfections in the measured film. The maximum difference between the two materials is seen in Figure 6d and Figure 6e, where the resonance frequency peaks are higher than in any other image. It is important to notice that the images in Figure 6c–f were acquired over a period of approximately 3 h using S-AFAM, while the same result using RT-AFAM would have taken more than 8 h with lower resolution as seen in Figure 6b.

**Figure 7:**
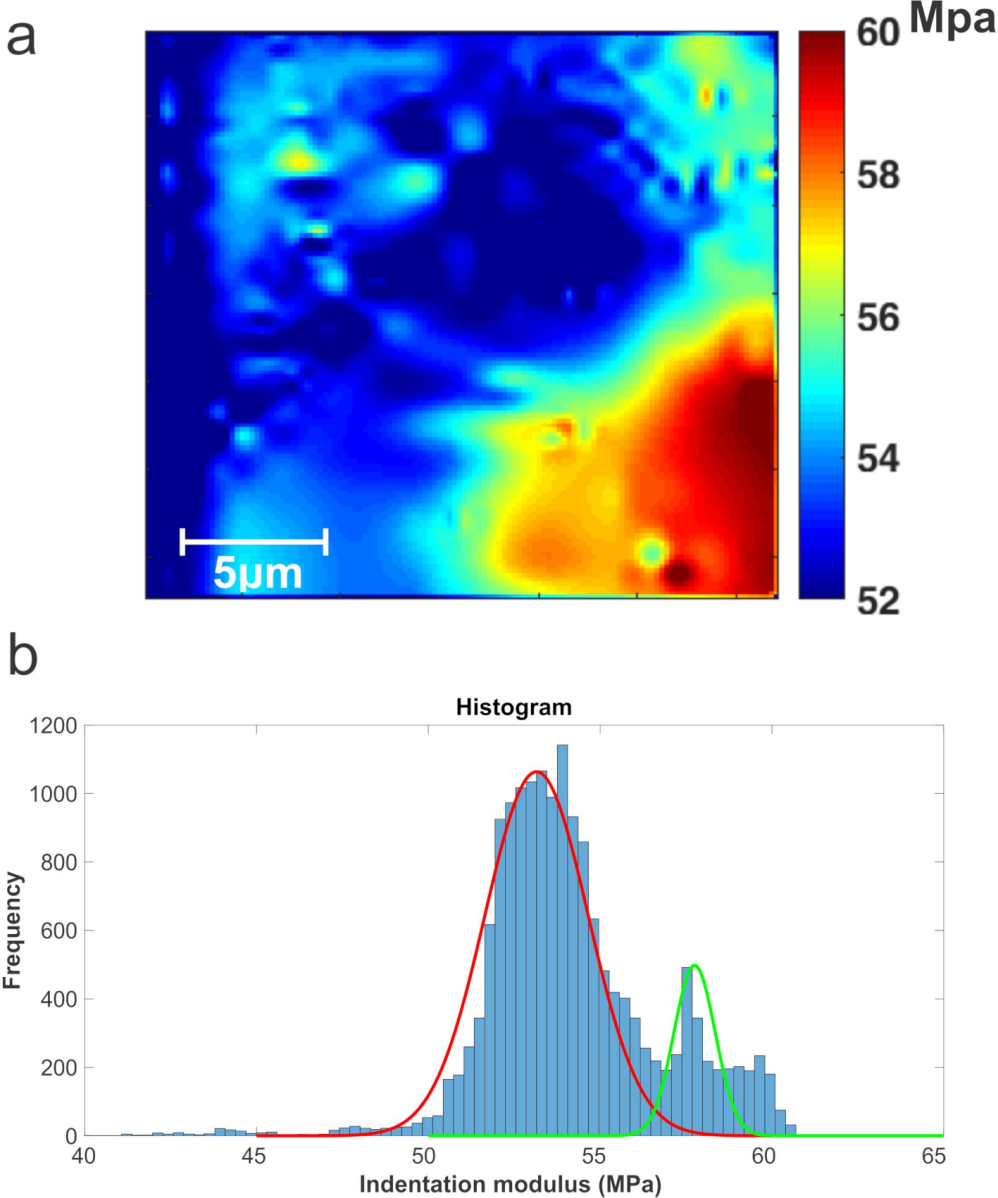
Results for a graphite film on a glass substrate: a) indentation modulus mapping and b) histogram for the mapping.

Finally, it is well known that the tip–sample interaction provides information about the contact stiffness, which is the product of effective contact and indentation modulus [9–10,19,38–46]. Using *k* = 2aE^*^ and E^*^ = (1/*M*_tip_ + 1/*M*_sample_)^−1^, where *a* ≈ 11 nm, which was obtained using the methodology in [47], *M*_tip_ = 170.33 GPa [37] and the proposed model, an indentation modulus mapping is obtained (Figure 7). This mapping was computed using the results shown in Figure 6c–f and the database shown in Figure 5.

In Figure 7a, a higher difference between glass and graphite film can be seen than in the RT-AFAM images. Even though the difference is very small, S-AFAM can detect the frequency shifts with higher resolution. The corresponding histogram is shown in Figure 7b. This difference is due to the contribution of the glass substrate and the low thickness of the graphite film. The indentation modulus values are 53.15 MPa for the glass substrate and 57.875 MPa for the graphite film. These results agree with the reports in literature [48].

The results make S-AFAM suitable for non-homogeneous materials in which the local mechanical properties of the materials yield similiar resonance frequencies. The use of white noise excitation perturbs all resonance frequencies at the same time, enabling the extraction of all resonant modes in one measurement.

Even though the deflection signal from the photodiodes is weak, when it is Fourier transformed, the amplitude increases significantly in the Fourier domain. This is possible due to the following Fourier transform property [26,32]

[29] f(t)↔F(ω),

for a real constant *a*,

[30] $$f\left(at\right)↔\frac{1}{\left|a\right|}F\left(\frac{\omega }{a}\right),$$

when |*a*| *<* 1, it is possible to measure higher resonance frequencies without losing frequency resolution.

Theoretically, a white-noise signal features an infinitely flat bandwidth, which is impossible to generate [35,49]. Fortunately, it can be generated in approximation using a waveform function generator. This makes the white-noise signal an ideal tool to extract all system dynamics instantaneously. It does not require a time excitation such as frequency sweep [20], i.e., it is a persistent excitation [23]. Additionally, the white-noise energy is lower than any other conventional signal, which avoids either electrical damage to the piezoelectric actuators or physical damage to the sample. It is noteworthy that S-AFAM can be enhanced when a more capable instrumentation is used, making the FFT computation faster, and when a better white-noise signal generator with a richer packet of frequencies is used, i.e., a thermionic diode.

## Conclusion

S-AFAM can provide more information about, e.g., aggregates, grain limits and mechanical stress of grains with similar local mechanical properties, which makes these properties difficult to discern by using conventional techniques. This is possible because a white-noise excitation enables the extraction of more information about the tip–sample interaction than any other kind of signal. Additionally, S-AFAM does not rely on a resonance frequency as other conventional techniques do. It uses reduced and optimal instrumentation, where the latter does not require a lock-in amplifier so that the signal from the photodiodes is not affected by the time constant of the lock-in amplifier. The extraction of resonant modes is obtained from one measurement, and the stored data is minimized, leading to less time needed for the measurement.

The results indicate that many contact resonance frequencies correspond to one indentation modulus value, while many values of indentation modulus would have been obtained by using other conventional techniques. For this reason, it is important to have more than one vibrational mode of the tip–sample interaction, since it provides quantitative knowledge about the contact stiffness, which is necessary for further analysis of local mechanical properties.

S-AFAM provides images not only of high frequency resolution but also of high depth resolution compared to conventional techniques. The latter lose resolution due to instrumentation and the kind of excitation signal used for experimental purposes. Using S-AFAM allows us to carry out in-depth analyses of local mechanical properties with a suitable model capable of making a relation between resonance frequency and indentation modulus.
